# Spatial signature of the photoelastic effect in the acoustic–plasmonic coupling revealed by space responsivity induced by polarized optical excitation

**DOI:** 10.1515/nanoph-2023-0701

**Published:** 2024-01-31

**Authors:** Zhiying Xia, Yang Zhang, Ruijie Hou, Bin Xu, Bin Ni, Jamie Jiangmin Hou, Lianping Hou, Xuefeng Liu, Jichuan Xiong

**Affiliations:** School of Electronic and Optical Engineering, Nanjing University of Science and Technology, Nanjing 210094, P.R. China; Department of Medicine, University of Cambridge, Hills Road, Cambridge, CB2 0QQ, UK; James Watt School of Engineering, University of Glasgow, Glasgow, G12 8QQ, UK

**Keywords:** space signature, localized surface plasmonic resonance, gold nanosphere, acoustic–plasmonic

## Abstract

Acoustic–plasmonic coupling in metallic nanoparticles can significantly alter their optical absorption and scattering characteristics. However, almost all previous investigations on acoustic–plasmonic coupling so far have been focused on the spectral response of particles in a vacuum. In this report, a spatial photon scattering mode taking count in the acoustic–plasmonic coupling of individual gold nanoparticle (GN) on a silicon substrate under ultrasonic influence was presented. The acoustic–plasmonic is visualized with parametric images with spatial scattering patterns of the particle under the excitation of polarized light along the Poincare’s equatorial trajectory. The ultrasonic sources can be sensitively extracted from the parametric sin*δ* images, providing clear evidence of the extremely weak influence of ultrasound wave directivity on the spatial characteristics of the scattering of the particle through acoustic–plasmonic coupling. Experiment and simulation results reveal that, in general, the coupling is the strongest, when the maximum electric field (plasmon vibration mode) aligns with the ultrasonic propagation direction. This study provides a new angle to observe and deepen the understanding of the acoustic–plasmonic effect of nanoparticles, in addition to the conventional manner of investigation on their scattering spectra. It emphasizes the possibility of determining the spatial distribution of nanoparticles via photon state scattering when they are in a weakly oscillating environment, providing valuable guidance for future potential applications exploiting the acoustic–plasmonic effect of nanostructures.

## 1 Introduction

The optical properties of metal nanoparticles arise from the coupling between incident electromagnetic radiation and collective oscillation of conduction electrons, and the resulting local surface plasmon resonance is widely used in various fields, such as the detection of mechanical properties of the nearby fluid environment [1], virus detection [2], multifunctional drugs for tumor diagnosis and treatment [3], [4], surface-enhanced Raman scattering [5], [6], [7], etc.

The dimensions and morphology of nanoparticles, along with the coupling between nanoparticles and their surrounding media, have a fundamental impact on the spectra of plasmon [8]. There is inevitably some vibration in the particle itself or its immediate environment under optical excitation, so there is a significant and fundamental interest in exploring the interplay between acoustic vibration and local surface plasmon [9], [10]. Acoustic vibrations can be generated using an ultrafast pump laser pulse [11], [12], a wide range of loads on the substrate [13], or via piezoelectric phenomena [10], [14]. Wherein, the ultrafast vibrational dynamics of metal structures can be investigated by femtosecond pump-probe spectroscopy [15], [16] and subpicosecond laser pulses [17] to detect and identify the various vibration modes of nanoparticles. Similarly, the photothermal effect shall also affect the morphology of the nanoparticles and surrounding environment [18], [19], [20], [21], [22] and subsequently affect the localized surface plasmon resonance (LSPR) spectrum of the nanostructures.

In certain research, an adjustable LSPR spectrum was developed through acoustic–plasmonic coupling in GNs arrays induced by shear horizontal vibration, resulting from a piezoelectric phenomenon [14]. However, this investigation solely considers the influence of the displacements of GNs caused by acoustic vibration on the LSPR spectrum, disregarding the acoustic–plasmonic mechanism related to the volume variation of nanoparticles. Numerical investigations conducted by Aftab Ahmed et al. on the modulation of the optical response of silver nanoparticles through acoustic vibrations took into account both mechanisms [23]. Through simulation and experiment, they have verified that nanoparticles integrated into a uniform dielectric host matrix result in changes in the LSPR spectrum via both the volume mechanism and photoelastic coupling. Nonetheless, prior research primarily concentrated on spectral characteristics without adequate discussion of the spatial field distribution for nanostructures under the influence of vibrations. Furthermore, there was scant attention paid to the effect of ultrasound with diverse settings.

Recently, it has been reported that single-molecule fluorescence signals are tracked to extract specific frequencies and amplitude of acoustic vibrations, which has a sensitive dependence on the distance between the dye crystal violet and a GN [9]. Although this study provides a very sensitive way for the detection of acoustic vibrations at the nanometer scale, it is still relying on exploiting the spectrum of single-molecule fluorescence and spatial characteristics of plasmonic scattering influenced by the acoustic–plasmonic effect in nanoscale systems remains to be investigated.

**Figure 1:**
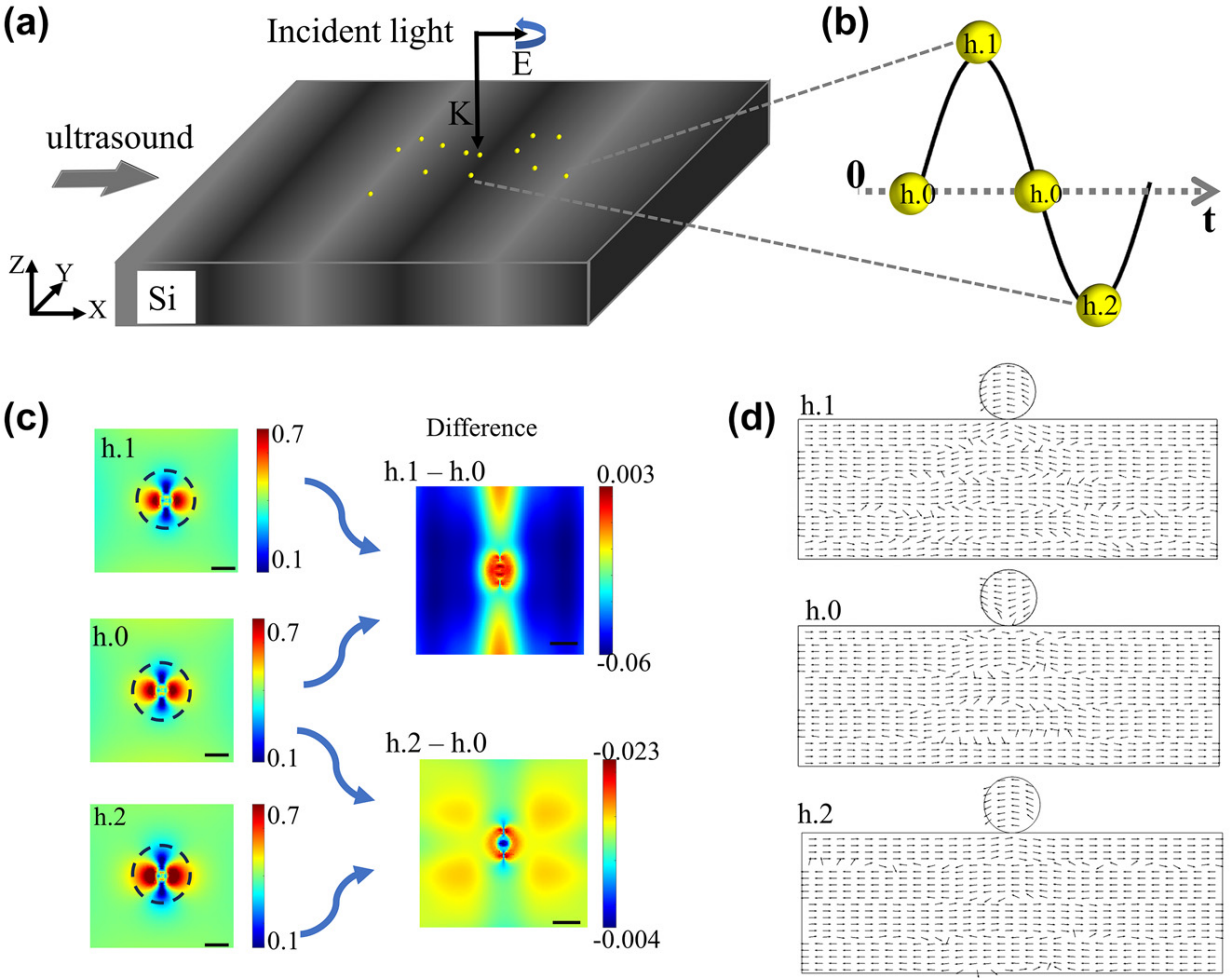
Characterization of gold nanospheres detection system for acoustic vibrations. (a) Schematic of the nano acoustic detector based on the photon state scattering of gold nanospheres. The nanospheres were attached to the silicon substrate, and acoustic vibration in the horizontal direction was propagating on the silicon substrate. (b) Three states of particles under ultrasound, in tension (case h.1), when the ultrasonic amplitude is 0 or in balance (case h.0), and when it is compressed (case h.2). (c) Sin*δ* parameter diagrams of gold nanospheres depicting differences among three acoustic vibration states, and the accompanying. Scale bar, 100 nm. (d) Electric field vector diagrams of the *X*–*Z* section in three ultrasound states.

In this paper, we reveal the spatial field distribution characteristics induced by the acoustic–plasma coupling phenomenon, which allows us to conclusively prove the existence of the acoustic–plasmonic effects in an individual nanostructure. The photon state parametric images, i.e., sin*δ* and *ϕ*, are applied to demonstrate the influence of mechanical oscillation of gold nanospheres adsorbed on the silicon substrate on its localized surface plasmonic resonance (see Figure 1), based on an indirect polarization imaging technique developed previously in the group [24], [25], [26].

The impact of ultrasonic waves with varied propagation directions and different moments on the scattering parameter images is discussed, which can assist in tracking ultrasonic sources sensitively. The experimental findings demonstrate that a difference in the scattered light field can be detected, even at an angle of 0.35° between the vectors connecting the particle and different sound sources, which can be verified by the simulations with the finite element method (COMSOL). Also, the LSPR spectrums were confirmed to shift due to the photoelastic mechanism and change with the polarization angle of the incident light. Ultrasound waves had a stronger influence on localized plasmonic effects when the propagation direction aligned with the incident light polarization. It is anticipated that the spatial scattering characteristics obtained through polarization parameter imaging will further aid in designing nanoparticle applications for various vibration environments.

## 2 Materials and methods

### 2.1 Sample preparation

In the following, we examined GNs attached to the silicon substrates. The stock solution of GNs was diluted to a concentration of 1 mg/mL with 100 mg/mL distilled water, and then 30 μL of the solution was dispensed onto the silicon substrates. The preparation of samples took place under ambient conditions. Piezoelectric ceramic (PZT) is attached to one side of the upper surface of the silicon substrate to facilitate acoustic vibrations. The PZT is linked to a signal generator via wires, delivering a sine wave with an amplitude of 10Vpp at a frequency of 10 MHz. On the opposite side, the other PZT is connected to an oscilloscope to observe the propagation of ultrasound waves on the silicon substrate.

### 2.2 Simulation setting

**Figure 2:**
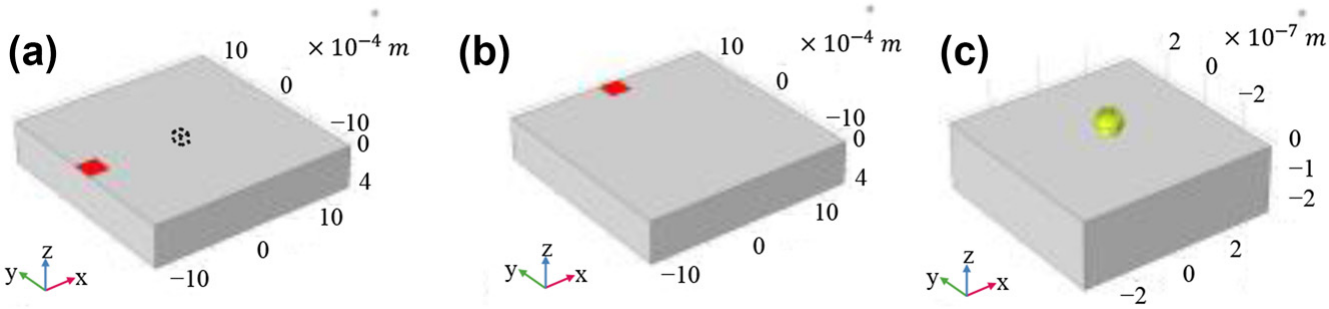
Models used in the simulation study. The dimensions of the silicon are 2.5 mm (length) and 2.5 mm (width), with a height of 0.5 mm. A GN with a 50 nm radius is centrally positioned within the silicon. Sine wave loads serve as the sound source. Red areas in (a) and (b) signify sound source locations on the left and above the upper surface of the silicon, respectively. In (c), the position of the GN relative to the silicon substrate is illustrated, with the GN centrally located above the silicon. The golden color represents the GN. The dimensions of silicon in (c) are 750 nm in width, 750 nm in length, and 250 nm in height. The position of (c) is demarcated by the black dotted line in the middle of (a), reflecting their size relationship.

The finite element software (COMSOL Multiphysics 5.5) is utilized to ascertain the optical response in various propagation directions of sound waves, by calculating its mechanical and optical responses. The propagation processes of waves are modeled using the Solid Mechanics Module. For the provision of ultrasonic waves, loads are employed instead of PZT, owing to its time-saving benefits. The loads were set in a sinusoidal form along the *Z*-axis, with an amplitude of 10Vpp and a frequency of 10 MHz, aligning with the established parameters of PZT in the experiment. The region of applied loads is situated on the top of the silicon substrate, as shown by the red area in Figure 2(a), with dimensions of 2.5 µm × 2.5 µm. The silicon substrate itself measures 2.5 mm × 2.5 mm × 0.5 mm, hosting a 100 nm GN positioned centrally, visible in the golden area in Figure 2(c). The mesh configuration employed is a free tetrahedral mesh, characterized by a maximum element size of 40 µm and a minimum element size of 2.5 nm.

To explore the influence of ultrasound in various propagation directions, models for sound waves propagating along the *X*-axis and *Y*-axis were established, respectively. These models are depicted by the red areas in Figure 2(a) and (b), respectively. The sound source is positioned either to the left or on the upper surface of the silicon.

The simulation of optical response utilizes the Electromagnetic Waves (Frequency Domain) Module. The refractive index (RI) of GN is sourced from Johnson and Christy, undergoing modulation influenced by elastic strains to facilitate the calculation of optical properties. In this section, the dimensions of the silicon substrate are specified as 750 nm × 750 nm × 250 nm, with the diameter of GN being 100 nm, as illustrated in Figure 2(c). Plane electromagnetic waves are employed at a distance of 300 nm above GN, and free tetrahedral meshes are configured with a maximum element size of 20 nm.

### 2.3 Optical measurements

The photon state parameter images (sin*δ* and *ϕ*) are acquired through the application of the polarization indirect microscopic imaging (PIMI) technology [27]. This involves exciting the localized plasmonic field of nanoparticles with the light along Poincare’s equatorial trajectory, detecting changes in the scattered photon state to visualize the anisotropic characteristics of the nanostructure. The resolution of this technique surpasses the diffraction limit of the traditional microscopy. The intensity of scattering in this approach significantly relied on the changes in the measured nearby-field refractive index.

A succinct description of the PIMI system is concluded here with additional information available in [27]. The system was assembled using a reflection microscope (BX51, Olympus) equipped with a combination of a PiA2400-17gm CCD (Balser) and a 100× objective to enhance sensitivity. A polarizer capable of modulating angles between 0 and 180° in increments of 18° was inserted within the illumination path. Interpolation was implemented to convert the image pixel size from 34.5 nm to 6.9 nm to achieve greater analysis accuracy.

### 2.4 Acoustic–plasmonic coupling

To investigate the optical response of nanoparticles to ultrasonic vibration, we utilize PZT as a source of ultrasonic waves instead of a pulsed laser because it exhibits piezoelectric phenomena and can disregard the photothermal effect. We thus concentrate solely here on the correlation between mechanical vibrations and plasmon resonances. For an individual GN, the motion of the particle caused by acoustic vibrations exclusively alters its location in space and has no impact on the connection between the particles and the substrate. Therefore, the paper excludes any consideration of horizontal particle displacements. The coupling between ultrasound waves and nanoparticles is identified as stemming from the elastic and volume effects. However, alterations to the LSPR spectrum will not occur when the volume remains constant despite shape changes [23], [28]. From the following simulated results, it is evident that the particle has minimal substrate contact, and its volume undergoes an insignificant change. This paper disregards the alteration caused by the volume effect and emphasizes the impact of the elastic effect.

To explain the optical properties of GNs, we apply the Lorenz-Drew model to articulate the dielectric constant of gold nanospheres:

(1) $$\varepsilon_{r}\left(\omega \right)=\varepsilon_{r}\left(\infty \right)+\omega_{p}^{2}\overset{M}{\underset{m=0}{\sum }}\frac{f_{m}}{\omega_{0,m}^{2}-\omega^{2}-j\omega \Gamma_{m}}$$

where plasmon frequency (*ω*_*p*_), oscillator strength (*f*_*m*_), linewidth (Γ_*m*_), and interband transition frequency (*ω*_0,*m*_) would undergo a transition under tensile or compressive strains. Thus, the shift can be evaluated $\omega_{0,m}^{\prime }=\omega_{0,m}+\frac{\xi_{m}\left(\gamma_{xx}+\gamma_{yy}+\gamma_{zz}\right)\left|e\right|}{\hbar }$ , where *ξ*_*m*_ is the deformation potentials, *γ* refers to the local strain, |*e*| represents the electron charge, and *ℏ* is the reduced Planck’s constant [23].

For the silicon substrate, the elastic effect of vibrations is primarily reflected in the alterations of the refractive index, which can be quantified as

(2) $$\begin{array}{ll} \left(\begin{array}{c} n_{xx}\\ n_{yy}\\ n_{zz}\\ n_{yz}\\ n_{xz}\\ n_{xy} \end{array}\right) & =\left(\begin{array}{c} n_{0}\\ n_{0}\\ n_{0}\\ 0\\ 0\\ 0 \end{array}\right)-\left(\begin{array}{cccccc} C_{1} & C_{2} & C_{2} & 0 & 0 & 0\\ C_{2} & C_{1} & C_{2} & 0 & 0 & 0\\ C_{2} & C_{2} & C_{1} & 0 & 0 & 0\\ 0 & 0 & 0 & C_{3} & 0 & 0\\ 0 & 0 & 0 & 0 & C_{3} & 0\\ 0 & 0 & 0 & 0 & 0 & C_{3} \end{array}\right)\left(\begin{array}{c} \sigma_{xx}\\ \sigma_{yy}\\ \sigma_{zz}\\ \sigma_{yz}\\ \sigma_{xz}\\ \sigma_{xy} \end{array}\right) \end{array}$$

where *C*_1_, *C*_2_, and *C*_3_ are stress-optic constants [29], [30], [31], [32], [33]. *n*_*ij*_ and *σ*_*ij*_ are the refractive index and stress, respectively, with *i* and *j* corresponding to *x*, *y*, and *z*.

Due to the nonuniform distribution (inhomogeneous) of stresses and their varying values in different directions (anisotropic) within the nanoparticle, the refractive indexes are also inhomogeneous and anisotropic.

## 3 Results and discussion

According to the acoustic wave excitation mode shown in Figure 2(a), the stress distribution at two seconds is selected as the moments when the nanoparticles were located at the valley and peak of the ultrasound wave on the basis of the sound pressure curve at the contact point (0,0,0) as the dilatation and compression state, when the pressure values are close after the sound waves were relatively stable. The sound waves propagating in the *X* direction are deemed as horizontal, with the ones that propagate in the *Y* direction being labeled as vertical.

The stress levels are approximately 10^7^ Pa, with the changes in dielectric permittivity caused by stress having real and imaginary parts on the order of 10^−12^ depending on the calculations utilized, while the reference [28] estimates the calculations to be on the order of 10^−1^. The modifications to the dielectric constant resulting from the excitation of sound waves by PZT are considerably reduced within this paper. With the given intensity of sound pressure, the largest displacements of nanoparticles are approximately 5 nm. This indicates that the changes in dielectric permittivity, due to the effects of displacement and shape change, may be disregarded in this paper. Subsequently, we examine the deviations of the extinction spectrum.

We first consider the influence of sound waves on the LSPR spectrum of GN at the valley and peak positions when it propagates horizontally, with the electromagnetic field’s polarization direction aligned with the *X*-axis’s positive direction in the simulation. Five distinct cases are proposed. Case h.0 entails GN subject to polarization illumination devoid of any acoustic vibrations, case h.* involves GN affected by vibrations traveling the horizontal direction at their crest (*.1) and trough (*.2), while case v.* involves GN exposed to ultrasound wave propagating vertically direction at their crest (*.1) and through (*.2).

**Figure 3:**
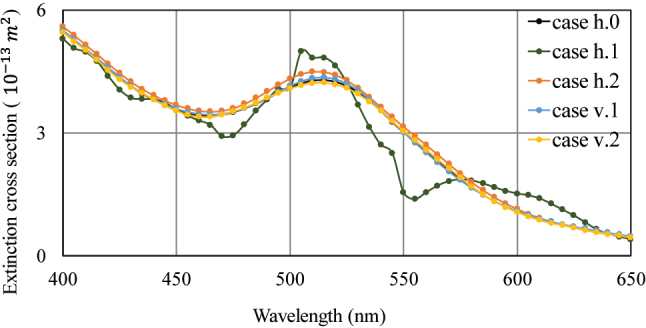
Optical extinction spectrum of GN under *X*-axis polarization spanning from 400 nm to 650 nm under various conditions of acoustic–plasmonic coupling for the simulation.

As depicted in Figure 3, the coupling of ultrasound vibrations and plasmonic resonance results in a notable blue-shift within the wavelength range of 500 nm to 550 nm, influencing the numerical value of the extinction cross section. Evidently, the values for case h.2 surpass those of case h.0, and the four curves maintain consistency above the 600 nm wavelength, except for case h.1.

However, there are exceptions. In case h.1, where vibration is at its peak in the horizontal direction, the resonance peak around 460 nm experiences a red-shift to 470 nm, causing a noticeable change. Furthermore, the resonance peak at 520 nm undergoes a blue-shift to 515 nm, and a new resonance peak emerges at 555 nm, as observed in Figure 3. The curve demonstrates that when the direction of sound wave propagation is consistent with the polarization direction of the light field, and the amplitude of the sound wave significantly impacts the tensile state of the ultrasound stress, it results in noticeable fluctuations the LSPR spectrum and could potentially add a resonance peak. The abrupt alteration observed in case h.1 could potentially be attributed by lager stress distribution, resulting in the anisotropy of refractive index distribution. In case h.1, the direction of sound wave propagation aligns with the polarization direction of the light field. Additionally, the sound pressure in this scenario is relatively higher compared to case h.2, leading to a sudden change in the curve. In contrast, in other cases, the curve remains smooth.

To further investigate the optical response and LSPR spectrum under the influence of ultrasound waves, the simulation results of GN induced by the Poincare equatorial trajectory of the incident light field are analyzed. Compare the extinction spectrum curves in multiple situations and select the 500 nm wavelength near the resonance peak of 515 nm, to conduct the subsequent research while keeping the extinction cross section numerical values as equal as possible.

**Figure 4:**
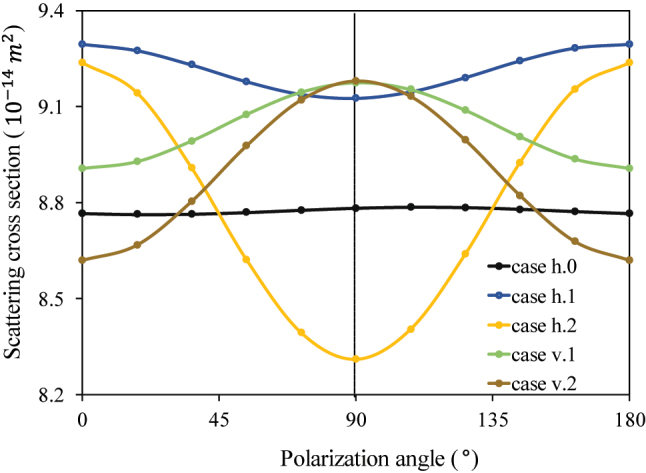
Influence of the acoustic–plasmonic coupling on the localized surface plasmon resonance (LSPR) scattering spectrum of GN at peak and valley values when subjected to ultrasound propagation along the horizontal and vertical directions for the simulation.

A 500 nm wavelength plane wave was propagated downward, perpendicular to the silicon substrate. Subsequently, we rotated the polarization direction angle in steps of 18° from 0° to 180° relative to the positive direction. In accordance with Figure 4, the scattering spectrum distributions of GN following acoustic–plasmonic coupling through two perpendicular acoustic wave propagation directions exhibit a sinusoidal pattern for half a period with the polarization direction of the incident light field, reaching the maximum values at 0° or 180°. When ultrasonic waves propagate vertically (Figure 4, cases v.1 and v.2) and the incident light is polarized along the *Y*-axis (with a polarization angle of 90°), the scattering spectrum is shifted more significantly in the horizontal direction. However, when the polarization direction is along the *X*-axis (i.e., with a polarization angle of 0°), the shift is smaller. It is also appropriate for horizontal ultrasound (Figure 4, case h.2) provided that the polarization direction aligns with the *X*-axis (0° the polarization angle). At this configuration, the maximum value of the offset caused by horizontally propagating ultrasonic waves can be achieved.

Two additional specific moments are selected to initiate the LSPR, and the outcomes exhibit nearly identical results as those presented in Figure 4. Despite the minuscule alteration in dielectric constant described in this paper, the augmented effect of acoustic–plasmonic is more conspicuous when the acoustic wave’s propagation direction corresponds to the polarization state of the incoming incident light field.

**Figure 5:**
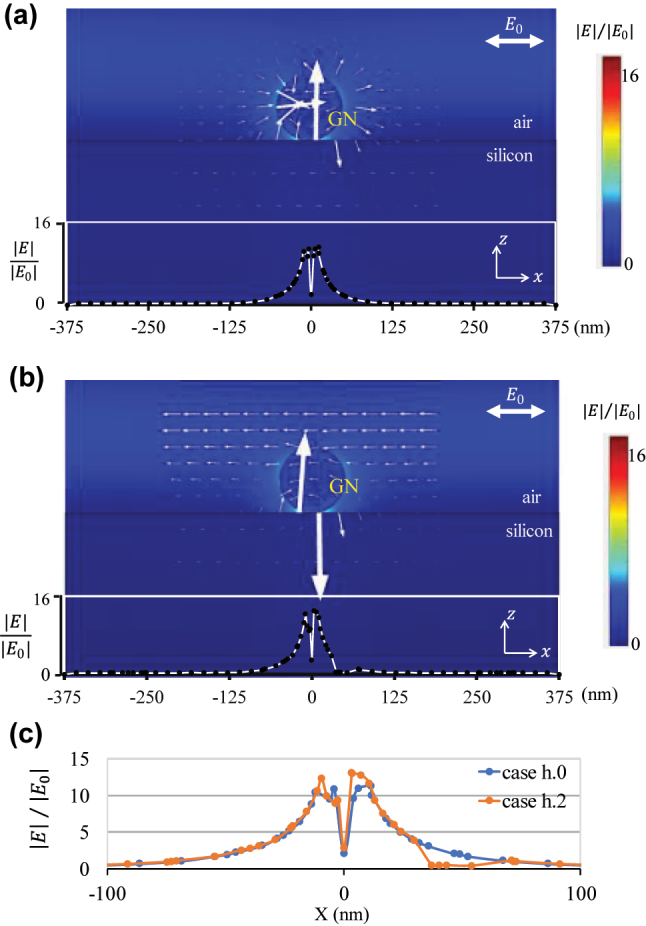
Electric fields distribution around the GN in the *X*–*Z* section for the simulation. (a) and (b) Depict the simulated distribution of the amplitude and the direction of the local electric field direction under case h.0 and case h.2, respectively. The arrows in the selected region indicate the electric field direction, and their lengths are an indication of the field length. The lower panels represent the magnitude of the electric field at the surface of the silicon substrate. (c) Shows the electric field amplitude on the surface of the silicon substrate in selected regions.

Figure 5 depicts a remarkable change in the electric field vector diagrams of GN in the *X–Z* section due to various ultrasound vibration conditions. This suggests the potential use of ultrasound waves in modifying the optical response of GN, resulting in the differences observed in the sin*δ* parameter diagrams. The electric field distribution indicates a noticeable change in the vector direction of gold particles on GN.

**Figure 6:**
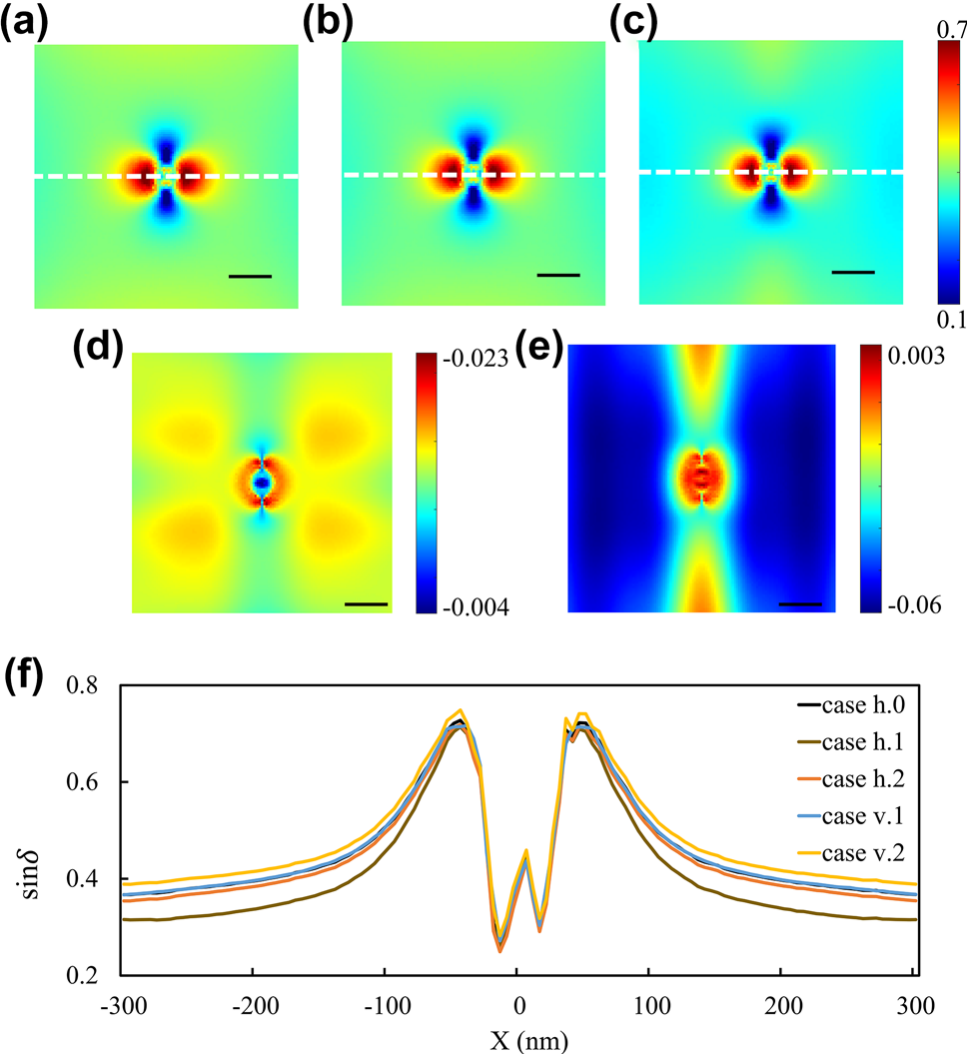
Sin*δ* images for the simulation. (a)–(c): PIMI images (sin*δ*) under the influence of case h.0 (a), case h.2 (b), and case h.1 (c) for the simulation. (d) The difference between (a) and (b) ((b) minus (a)), (e): the difference between (a) and (c) ((c) minus (a)), and (f): comparisons between (a) (b) and (c) along dashed white lines. Scale bar: 100 nm.

Photon state parametric images of the sample, *i.e.*, sin*δ*, are available for retrieval (refer to Figure 6). As changes are minimal, the light intensity profile curve is drawn at the dipole region of GNs exhibiting strong scattering (Figure 6(f)). There exists a four-tier scattering distribution, which consists of intense dipole scattering, mapped red region in Figure 6(a), and feeble dipole scattering in the blue region in Figure 6(a) surrounding GN. The scattering region initiates from the boundary of GN and spreads outward. Under the coupling of the acoustic vibrations and plasmonic resonance, the strong dipole scattering region can either increase (c) or decrease (b), and correspondingly, the areas of the weak scattering region in case h.2 are larger than those of case h.0. These changes are more apparent in diagrams (d) and (e). Figure 6(d) presents all negative values, indicating that the intensity of sin*δ* in case h.2 is lower than that in case h.0, while the (e) diagram displays that there are positive and negative values, representing that the scattering intensity in sin*δ* of case h.1 is slightly increased in the central region compared to case h.0. Weber contrast is employed to assess the contrast of the image. The calculation formula for contrast [34] is $WC=\left(I-I_{b}\right)/I_{b}$ , where *I* and *I*_*b*_ denote the intensity of the image and the background, respectively. The contrast of the image for case h.0 is 0.944, whereas those for case h.1 and h.2 are 1.244 and 0.988, respectively. The photoelastic effect induced by acoustic–plasmonic coupling has been found to effectively enhance the contrasts of the images.

**Figure 7:**
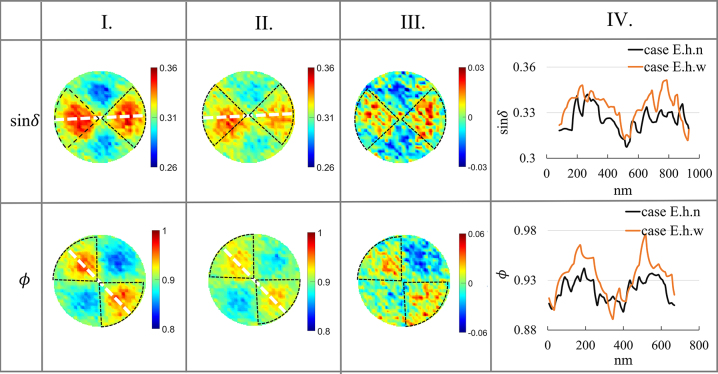
PIMI images (sin*δ* and Φ) for the experiment. I.: Under the influence of ultrasound in the horizontal direction (case E.h.w), II.: without ultrasound (case E.h.n), III.: the difference between I. and II., and IV.: the comparisons between I. and II. along dashed white lines. The size of the image, diameter: 940 nm.

**Figure 8:**
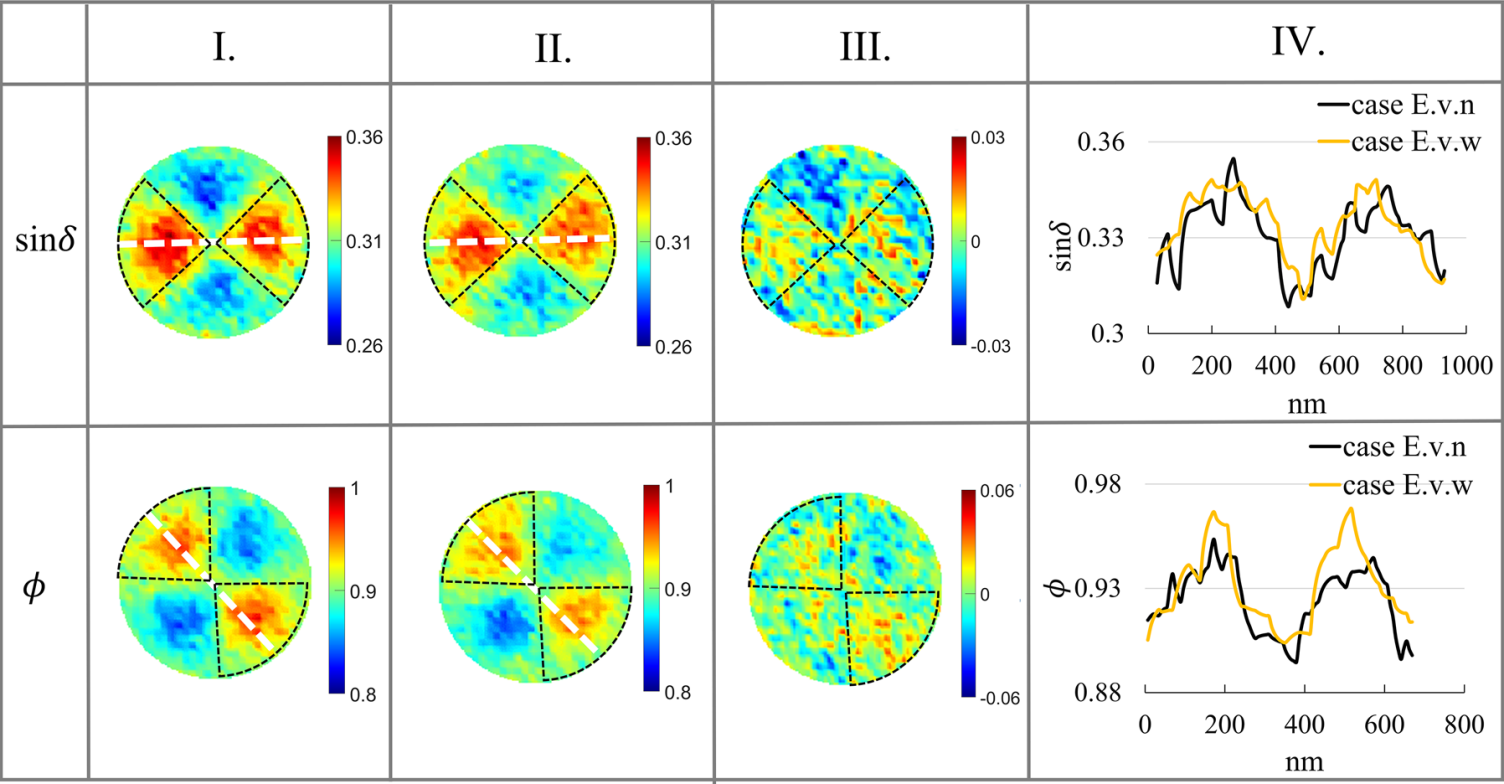
PIMI images (sin*δ* and Φ) for the experiment. I.: Under the influence of ultrasound in the vertical direction (case E.v.w), II.: without ultrasound (case E.v.n), III.: the difference between I. and II., and IV.: the comparisons between I. and II. along dashed white lines. The size of the image, diameter: 940 nm.

The experiment shows that ultra-weak spatial distribution is present in the parameter images. Figures 7 and 8 highlight the strong coupling region, indicated by black circles.

It has been discovered that the acoustic wave has minimal influence on the plasmon spatial scattering images for both sin*δ* and *ϕ* images. When two kinds of sound waves propagate in different directions, the polarization parameter images (sin*δ*) remain weak in term of weak spatial characteristic images after acoustic–plasmon coupling. This is evident from comparing the residual amount. Upon analysis of Figures 7 and 8, it is apparent that the enhancement effect of ultrasonic waves in the horizontal direction is more considerable compared to the vertical direction. This observation confirms that the enhancement effect of acoustic–plasmonic coupling is more pronounced when the propagation direction of the acoustic wave is consistent with the polarization state of the detection light field.

Different types of ultrasound waves have been produced in a sinusoidal form by variation of the frequency and amplitude of the PZT for the experiment. These results demonstrate that the scattering pattern under the influence of acoustic waves of GN differs compared to that under the electromagnetic fields alone. Additionally, the sin*δ* diagrams are slightly enhanced under the influence of ultrasound waves, whether under the influence of horizontal or vertical ultrasound waves. But unfortunately, the obvious regular patterns have not yet found from the existing experiments that have been performed. The possible reasons we judge are that, in the experiments, continuous sinusoidal signals are applied to the PZT, which generates continuous sound waves.

**Figure 9:**
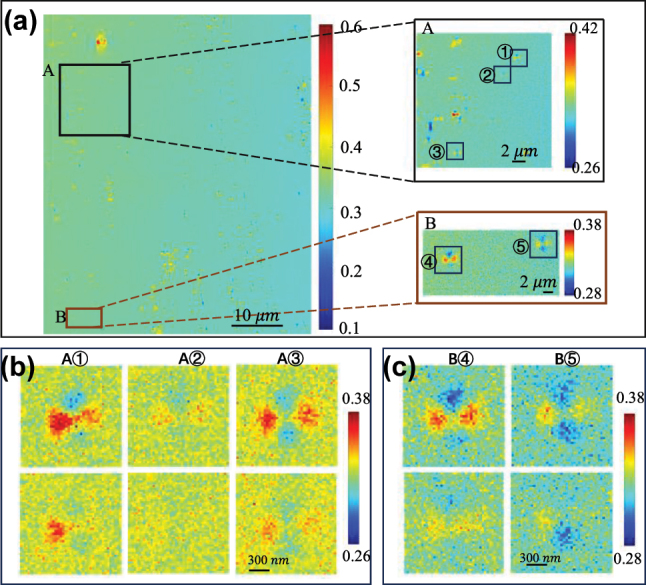
PIMI images (sin*δ*) for the experiment. (a) PIMI images (sin*δ*) obtained by using a 100× objective lens under bright field illumination under the influence of ultrasound in the horizontal direction. The right-hand sides display the magnified outcomes of the left square regions in order. (b) and (c) PIMI images (sin*δ*) for five individual GN in (a) respectively, from top to bottom: under the influence of ultrasound (case E.h.w), and the second row is without ultrasound (case E.h.n).

To confirm the sensitivity tracking of ultrasonic sources in sin*δ* images, the regions at both ends of the image over a field of view (FOV) of 53 μm × 63.3 μm are selected, as illustrated in Figure 9. The calculated displacement of the particles in Figure 9 is less than 2.3 nm. In consideration that the GN may not be at the same height during preparing the sample, three and two separate particles are selected in region A and region B, respectively. Figure 9(b) and (c) provide additional evidence that ultrasound vibrations enhance the contrast of sin*δ* images. The maximum value, minimum value, and peak-to-peak value of five individual GNs are extracted in Table 1 and 2. After calculation, the mean value of peak-to-peak values of the three particles was found to be 0.08183 in area A and 0.0827 in area B. It can be inferred that it is possible to differentiate the scattering field distribution of GN with differing distances from the sound source in this FOV. The focal region of interest in the experiment is positioned 10 mm distant from the sound source, indicating an included angle of about 0.34° between the nanoparticles and the sound source. This makes it feasible to differentiate the position of the sound source based on scattering diagrams of the nanoparticles.

**Table 1:** Comparison of sin*δ* images of nanoparticles in different positions under the influence of ultrasound in the horizontal direction for the experiment.

	A①	A②	A③	B④	B⑤
Peak-to-peak	0.0944	0.0684	0.0827	0.0882	0.0772
Average	0.08183			0.0827	

**Table 2:** Comparison of sin*δ* images of nanoparticles in different positions without ultrasound for the experiment.

	A①	A②	A③	B④	B⑤
Peak-to-peak	0.0671	0.0503	0.0749	0.0585	0.055
Average	0.0641			0.05675	

The sensitivity of sound sources of scattering distribution of GN is illustrated by simulation. Based on the model in Figure 2(a), the GN is moved 7.5 μm in the positive *Y* direction (case m), resulting in an angle of 0.34° between the GN and the sound source. The comparison results are statistically listed in Table 3. Following the above discussion, the trend in curve changes at valley value (case *.2) aligns with the expectations. Hence, we compare the results at valley value across three propagation directions. The scattering sin*δ* diagrams exhibit sensitivity toward sound sources. When the propagation direction of a sound wave is consistent with the polarization direction of the incident light field, the peak-to-peak value of the image is at its minimum of 0.7134. With the polarization direction of the sound wave and the incident light field increasing, the peak-to-peak value also increases to 0.7149. The maximum peak-to-peak value of 0.7269 is reached when the sound wave is perpendicular to the polarization direction of the incident light field.

**Table 3:** Comparison of sin*δ* images under different states for the simulation.

	Case h.0	Case h.2	Case m.2	Case v.2
Peak-to-peak	0.7059	0.7134	0.7149	0.7269

## 4 Conclusions

In conclusion, we investigate the spatial characteristics of scattering photons from individual nanoparticles under the influence of acoustic–plasmonic coupling, utilizing the polarization parametric visualization of the plasmonic scattering. The relations between the ultrasonic field and polarized excitation field along the equatorial track on the Poincare sphere are described qualitatively. Ultraweak acoustic–plasmonic effect in nanostructures was extracted from the spatial pattern of parametric imaged and even weaker directivity influence from different ultrasonic sources was extracted from the plasmonic scattering.

Due to the limited response of the sin*δ* scattering diagrams to sound waves in various propagation directions, it has been challenging to quantify the positional information of the sound source. We hope that future advancements will enable the extraction of sound wave propagation directions based on GNs positioned at defined locations on the silicon substrate, allowing for the accurate localization of the sound source.

Parametric sin*δ* images reveal sensitivity to the local plasmonic scattering field with acoustic–plasmonic coupling. In addition, the enhancement effect of acoustic–plasmonic coupling becomes more apparent when the propagation direction of the acoustic wave is consistent with the polarization direction of the incident light field. The findings verify that spatial scattering characteristics of nanoparticles can be used as sensitive signatures of ultraweak acoustic-coupling in individual nanostructures. They also open opportunities for investigating acoustic–plasmonic phenomena of nanostructures in a new dimension instead of widely exploited scattering spectra, which implies a practical and efficient sensing technique in various new applications of nano acoustic structures and devices based on acoustic–plasmonic phenomena.

## References

[1] Yu K., Major T. A., Chakraborty D., Devadas M. S., Sader J. E., Hartland G. V. (2015). Compressible viscoelastic liquid effects generated by the breathing modes of isolated metal nanowires. *Nano Lett.*.

[2] Zhang H. (2021). Photon scattering signal amplification in gold-viral particle ligation towards fast infection screening. *IEEE Photonics J.*.

[3] Nanjunda S. B. (2022). Emerging nanophotonic biosensor technologies for virus detection. *Nanophotonics*.

[4] Lim J. (2015). Identification of newly emerging influenza viruses by surface-enhanced Raman spectroscopy. *Anal. Chem.*.

[5] Tahir M. A., Dina N. E., Cheng H., Valev V. K., Zhang L. (2021). Surface-enhanced Raman spectroscopy for bioanalysis and diagnosis. *Nanoscale*.

[6] Sun A. Y. (2021). Diverse substrate-mediated local electric field enhancement of metal nanoparticles for nanogap-enhanced Raman scattering. *Anal. Chem.*.

[7] Zuo Z. (2022). Multiple plasmon couplings in 3D hybrid Au-nanoparticles-decorated Ag nanocone arrays boosting highly sensitive surface enhanced Raman scattering. *Nano Res.*.

[8] Li J. (2011). Effect of shape, height, and interparticle spacing of Au nanoparticles on the sensing performance of Au nanoparticle array. *Chin. Opt. Lett.*.

[9] Xie M. (2022). Ultrasensitive detection of local acoustic vibrations at room temperature by plasmon-enhanced single-molecule fluorescence. *Nat. Commun.*.

[10] Yakovlev V. V. (2013). Ultrasensitive non-resonant detection of ultrasound with plasmonic metamaterials. *Adv. Mater.*.

[11] Crut A., Maioli P., Fatti N. D., Vallée F. (2015). Acoustic vibrations of metal nano-objects: time-domain investigations. *Phys. Rep.*.

[12] Ahmed A. (2022). Low-frequency oscillations in optical measurements of metal-nanoparticle vibrations. *Nano Lett.*.

[13] Zhang P., Wang Y., He D., Xiao M. (2019). Numerical analysis of simultaneous measurement of the refractive index and the pressure utilizing the mode splitting in a single-opening microring resonator. *Opt. Commun.*.

[14] Firmansyah T., Wibisono G., Rahardjo E. T., Kondoh J. (2022). Reconfigurable localized surface plasmon resonance spectrum based on acousto-dynamic coupling in arrays gold nanoparticles induced by shear horizontal vibration. *Appl. Surf. Sci.*.

[15] Voisin C., Christofilos D., Fatti N. D., Vallée F. (2002). Environment effect on the acoustic vibration of metal nanoparticles. *Phys. B*.

[16] Mante P., Belliard L., Perrin B. (2018). Acoustic phonons in nanowires probed by ultrafast pump-probe spectroscopy. *Nanophotonics*.

[17] Hartland G. V. (2006). Coherent excitation of vibrational modes in metallic nanoparticles. *Annu. Rev. Phys. Chem.*.

[18] Hu M., Wang X., Hartland G. V., Mulvaney P., Juste J. P., Sader J. E. (2003). Vibrational response of nanorods to ultrafast laser induced heating: theoretical and experimental analysis. *J. Am. Chem. Soc.*.

[19] Baffou G., Quidant R., Girard C. (2009). Heat generation in plasmonic nanostructures: influence of morphology. *Appl. Phys. Lett.*.

[20] Baffou G., Rigneault H. (2011). Femtosecond-pulsed optical heating of gold nanoparticles. *Phys. Rev. B*.

[21] Ma C. (2021). Chiral optofluidics with a plasmonic metasurface using the photothermal effect. *ACS Nano*.

[22] Dixon A. J., Hu S., Klibanov A. L., Hossack J. A. (2015). Oscillatory dynamics and in vivo photoacoustic imaging performance of plasmonic nanoparticle-coated microbubbles. *Small*.

[23] Ahmed A., Pelton M., Guest J. R. (2017). Understanding how acoustic vibrations modulate the optical response of plasmonic metal nanoparticles. *ACS Nano*.

[24] Liu W. (2021). Characterization of deep sub-wavelength nanowells by imaging the photon state scattering spectra. *Opt. Express*.

[25] Jin X. (2023). Sub-wavelength visualization of near-field scattering mode of plasmonic nano-cavity in the far-field. *Nanophotonics*.

[26] Liu W. (2021). Polarization multi-parametric imaging method for the inspection of cervix cell. *Opt. Commun.*.

[27] Jin X. (2021). Label-free sensing of virus-like particles below the sub-diffraction limit by wide-field photon state parametric imaging of a gold nanodot array. *Nanoscale Adv.*.

[28] Saison-Francioso O., Leveque G., Akjouj A. (2020). Numerical modeling of acousto-plasmonic coupling in metallic nanoparticles. *J. Phys. Chem. C*.

[29] Huang M. (2003). Stress effects on the performance of optical waveguides. *Int. J. Solids Struct.*.

[30] Sapozhnikov O. A., Maxwell A. D., Bailey M. R. (2020). Modeling of photoelastic imaging of mechanical stresses in transparent solids mimicking kidney stones. *J. Acoust. Soc. Am.*.

[31] Zhu J. (2010). On-chip single nanoparticle detection and sizing by mode splitting in an ultrahigh-Q microresonator. *Nat. Photonics*.

[32] Djemia P., Bouamama K. (2013). Ab-initiocalculations of the photoelastic constants of the cubic SiC polytype. *J. Phys.: Conf. Ser.*.

[33] Welkowsky M., Braunstein R. (1972). Interband transitions and exciton effects in semiconductors. *Phys. Rev. B*.

[34] Li W., Lu C., Zhang J. (2013). A lower envelope Weber contrast detection algorithm for steel bar surface pit defects. *Opt. Laser Technol.*.

